# The Palliative Care in the Metastatic Spinal Tumors. A Systematic Review on the Radiotherapy and Surgical Perspective

**DOI:** 10.3390/life12040571

**Published:** 2022-04-12

**Authors:** Giuseppe Roberto Giammalva, Gianluca Ferini, Fabio Torregrossa, Lara Brunasso, Sofia Musso, Umberto Emanuele Benigno, Rosa Maria Gerardi, Lapo Bonosi, Roberta Costanzo, Federica Paolini, Paolo Palmisciano, Giuseppe Emmanuele Umana, Rina Di Bonaventura, Carmelo Lucio Sturiale, Domenico Gerardo Iacopino, Rosario Maugeri

**Affiliations:** 1 Neurosurgical Clinic, AOUP “Paolo Giaccone”, Post Graduate Residency Program in Neurologic Surgery, Department of Biomedicine Neurosciences and Advanced Diagnostics, School of Medicine, University of Palermo, 90127 Palermo, Italy; fabiotorregrossa00@gmail.com (F.T.); brunassolara@gmail.com (L.B.); sofiamusso.sm@gmail.com (S.M.); umbertobenigno@gmail.com (U.E.B.); rosamariagerardimd@gmail.com (R.M.G.); lapo.bonosi@gmail.com (L.B.); robertacostanzo3@gmail.com (R.C.); federicapaolini94@gmail.com (F.P.); gerardo.iacopino@gmail.com (D.G.I.); rosario.maugeri1977@gmail.com (R.M.); 2 Department of Radiation Oncology, REM Radioterapia srl, 95125 Catania, Italy; gianluca.ferini@grupposamed.com; 3 Department of Neurosurgery, Cannizzaro Hospital, Trauma Center, Gamma Knife Center, 95125 Catania, Italy; paolo.palmisciano94@gmail.com (P.P.); umana.nch@gmail.com (G.E.U.); 4 Department of Neurosurgery, Fondazione Policlinico Universitario “A. Gemelli” IRCCS, Università Cattolica del Sacro Cuore, 00168 Rome, Italy; rina.di.bonaventura@hotmail.it (R.D.B.); carmelo.sturiale@policlinicogemelli.it (C.L.S.)

**Keywords:** spinal metastasis, palliative care, quality of life, radiotherapy, radiofrequency ablation, vertebral augmentation, spinal cord stimulation

## Abstract

Spine represents the most common site for metastatic disease involvement. Due to the close relationship between the spinal cord and critical structures, therapeutical management of metastatic spinal cord disease remains challenging. Spinal localization can lead to neurological sequelae, which can significantly affect the quality of life in patients with a limited life expectancy. The authors conducted a systematic literature review according to PRISMA guidelines in order to determine the impact of the most updated palliative care on spinal metastases. The initial literature search retrieved 2526 articles, manually screened based on detailed exclusion criteria. Finally, 65 studies met the inclusion criteria and were finally included in the systematic review. In the wide scenario of palliative care, nowadays, recent medical or surgical treatments represent valuable options for ameliorating pain and improving patients QoL in such this condition.

## 1. Introduction

Bone is one of the most common sites of metastases from multiple types of cancers and spinal metastases represent up to 80% of bone metastases [1,2]. They can occur 20 times more often than primary neoplasms of the spine, causing pain, pathological fractures, spinal cord and nerve roots compression and neurological impairments [1,3,4].

Over the years, incidence of spinal metastases have increased due to improved diagnostics and life expectancy of cancer patients [5,6].

In this regards, preservation of patient Quality of Life (QoL) is mandatory event in the latter stage cancer course. However, spinal metastases may determine spinal instability, with movement-related pain, symptomatic or progressive deformity, and/or neural impairments.

Since the short life expectancy of affected patients, an early diagnosis should be achieved and a prompt treatment is advised in order to ensure a quick pain relief; nevertheless, suspecting early spinal metastases through radiography is difficult and diagnosis is often tardive [7].

For this reason, spinal metastases are often addressed to palliation through several conservative and surgical treatments.

In recent times, several advancements in oncological treatment have resulted in a longer survival of patients with spinal metastases. Therefore, palliative treatments even more aim to improve patient’s QoL since the longer life expectancy. 

Unfortunately, there is still no consensus on the most effective treatment to improve patient’s QoL and palliative treatments have to be tailored on patient’s status and life expectancy. They both are assessed through several prognostic scores specifically designed for spinal metastatic patients. 

Since the balancing between surgical risks and life expectancy, patients are often addressed to palliative care, which nowadays can rely on many different non-operative or minimally invasive treatments.

After an extensive systematic review on literature, we therefore report an updated overview of the latest palliative treatments currently used in the management of symptomatic spinal metastases, in order to provide an insightful descriptive overview on the latest available treatments and to point out their efficacy in pain relief and the subsequent improvement of patient’s QoL.

## 2. Materials and Methods

A systematic review of the literature was performed in order to assess the current treatments for the palliation of spinal metastases. A comprehensive search according to PRISMA guidelines was conducted on PubMed and MEDLINE in September 2021. Databases were queried for the following search strings: “spinal metastasis AND palliative care”, “spinal metastasis AND palliative treatment”, “vertebral metastasis AND palliative treatment”, “vertebral metastasis AND palliative care”. In addition, only articles published within the last 5 years were considered. 

Screening of titles and abstracts, as well as eligibility assessment of full-text articles, was conducted according to the topic of this review. 

Exclusion criteria were the following: non-English studies, publishing date before January 2017; studies discussing only vertebral fixation and posterior decompression; studies analyzing side effects of palliative approaches without investigating the outcome of each therapeutical approach; meta-analyses, reviews, surveys, preclinical studies, letters to the editor, commentaries, isolated case reports. 

Articles discussing the clinical outcome, survival, and quality of life of patients undergoing palliative treatment for spinal metastases were included.

## 3. Results

**Figure 1:**
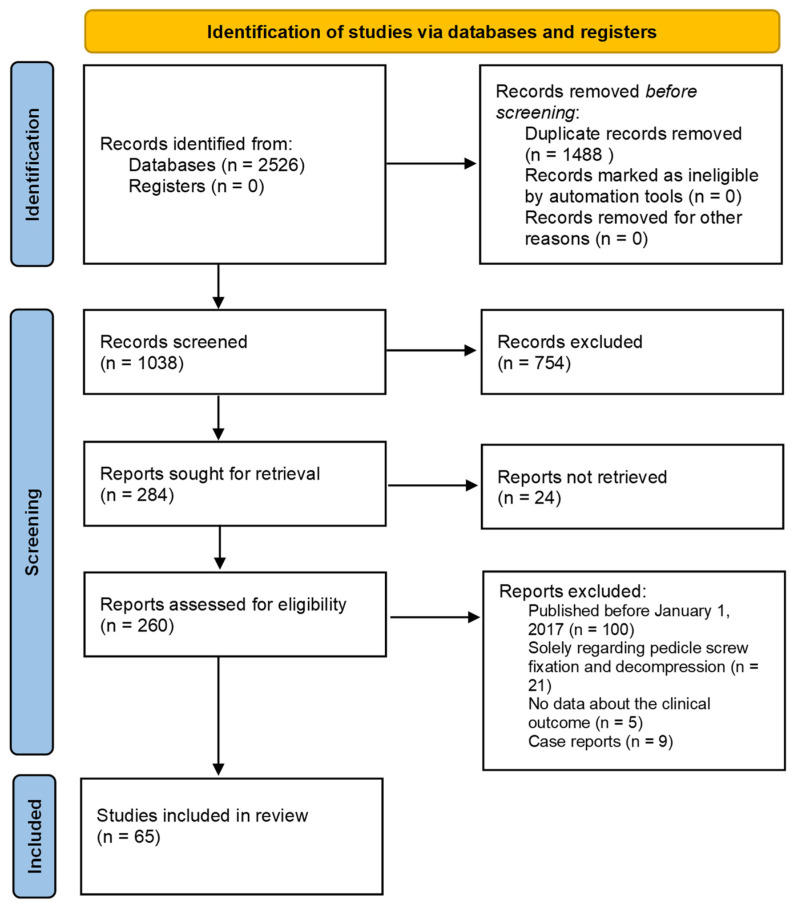
This is a figure. Schemes follow the same formatting.

We obtained a review of the most updated evidence of the current palliative management of spinal metastases through a systematic and extensive literature search strategy according to PRISMA guidelines (Figure 1). The initial database queries yielded 2526 articles, of which 1488 were duplicates. The remaining 1038 unique records were manually screened and 754 were excluded since they did not meet our inclusion criteria. Among the remaining articles, 24 more articles were excluded due to the lack of full-text. Subsequently, 260 articles were assessed for eligibility and 160 more studies were excluded because according to our exclusion criteria. In particular, 100 articles published before January 2017, 21 studies regarding only vertebral fixation and decompression, 5 studies with no sufficient data about clinical outcome, and 9 case reports were excluded. Finally, through our comprehensive search and screening strategy 65 studies were included in this systematic review. 

## 4. Discussion

In recent decades, important progress have been made in the medical treatment of oncological diseases resulting in considerable increase of survival time. Therefore, the management of cancer patients focuses nowadays on every potential way to improve their quality of life (QoL). The spine is a common metastatic location and this constitute a key element not just for the tumor evolution but also for the patient’s prognosis [8]. Vertebral location carries real risks of further neurological complications, in the setting of an already limited life expectancy and psychological deterioration. 

Spontaneous pathological fractures due to unstable and weak vertebrae can occur, and most patients with vertebral metastases present intractable pain significantly affecting their QoL [9]. Patients who present isolated vertebral metastases at an early stage may be treated with radical resection [8]. With the exception of such isolated situations, the treatment for vertebral metastases is usually palliation, aimed to pain relief and to the maintenance of neurological function. 

In an era of improved oncological treatments, therapeutic decisions for patients with vertebral tumors have become more complex, as patients survive long enough to experience morbidity not just from tumor progression but also from oncological therapies [10]. Currently, there is no consensus on the most effective treatment to improve QoL in patients with vertebral metastases. Generally, decisions regarding the surgical treatment are based on both the balancing between surgical indication and risk and life expectancy; hence, non-operative palliative care is often the best option for the treatment of spinal metastases [8]. As regard the treatment of pain, opioids represent the last but most effective treatment in order to achieved pain control [11,12,13]. Nonetheless, opioid intake has major side effects, mental and/or physical dependence and tolerance leading to loss of effectiveness. On the other hand, multidisciplinary approach to the patient with spinal metastases is nowadays considered the best practice since it provides appropriate staging before planned interventions and customized treatment for each specific condition [10]. Several studies demonstrated also an impact on reducing health care expenditures, increasing affordable insurance coverage, and improving qualitative changes in psychological and functional outcomes of care therapy [14,15].

Cancer-associated pain shares multifactorial and complex causes, and these are likely to vary with tumor-related and patient-related factors. Since this complex behavior, the treatment of pain related to spinal metastasis may require different and personalized techniques. In the whole scenario of palliative treatments, we resume the updated evidences on the most recent treatments for the palliative care of spinal metastases. 

### 4.1. Prognostic Scoring Systems for Spinal Metastases

Since the great variability in overall clinical presentation of patients with spinal metastases, reliable tools are required to precisely define the best palliative treatment for each patient. In order to reduce the subjectivity of clinical evaluation and to precisely estimate life expectancy, during the last decades several prognostic scoring systems have been proposed [16,17].

Among these, *Tokuhashi score*, *Tomita score*, *Bauer Scoring System* and *Van der Linden scoring system* are currently used to evaluate patient’s prognosis basing generally on systemic characteristics of primary tumor, spinal involvement and patient’s general performance status [18,19,20,21,22,23]; whereas *Katagiri score* takes into consideration a history of chemotherapy as key factor for prognostic evaluation [24] and *Oswestry Spinal Risk Index (OSRI)* is aimed to simplify the evaluation of patient prognosis considering two factors: type of primary tumor and general condition [25]. However, these scoring system are aimed to estimate overall life expectancy and not to define the type of treatment.

On the contrary, *Rades score* is strictly aimed to the evaluation of radiotherapy as palliative treatment for patients with spinal metastasis at an advanced stage and impending paralysis [26]; whereas *Spinal Instability Neoplastic Score (SINS)* is aimed only to assess the degree of spinal instability order to address the most convenient surgical intervention [6].

More recently, the variety of prognostic scoring systems has widened by the introduction of other scores with higher sensitivity and specificity in predicting long-term post-operative survival than the previous ones. In detail, *SORG (skeletal oncology Research Group) nomogram* and the *New England Spinal Metastasis Score (NESMS)* have been validated for the survival estimation of patients with spinal metastases. As regard the SORG nomogram, its clinical usefulness has been demonstrated since its capability of estimating 3 and 12 months individual survival probability of patients candidate to surgery through the evaluation of continuous variables such as hemoglobin levels, white blood cell count, age, previous systemic therapy, systemic metastases, multiple spinal metastases, primary tumor and performance status [27].

On the other and, NESMS has been recently validated as clinical tool capable of predicting 1-year morbidity and mortality of patients undergone surgery for spinal metastasis through the evaluation of preoperative modified Bauer score, ambulatory function and serum albumin. In particular, the higher the NESMS pre-operative score is, the higher the odd of survival is 1 year after the treatment [28,29].

### 4.2. Radiotherapy 

Radiotherapy is the most common palliative treatment for spinal metastases; its main purposes are palliation of pain, achievement of local tumor control, improvement of patient’s quality of life and prevention of adverse-related events, such as pathologic fractures and spinal cord compression. 

Several studies consider *conventional external beam radiation therapy (cEBRT*) as the primary treatment method for spinal metastases, both in oligometastatic and polymetastatic Spinal Tumors [30,31,32].

Generally, radiotherapy is effective for pain reduction and in maintaining skeletal integrity, while minimizing the occurrence of adverse related events such as pathological fractures. In facts, osteolytic metastases that have been successfully treated by RT subsequently appear normal or sclerotic on CT scan, due to re-ossification; thus, CT-follow up can be used to evaluate therapeutic outcomes after RT for bone metastases [30]. 

As regards pain control, it has been demonstrated a significant decrease of pain after RT up to 93% [31]. In case of vertebral metastases, pain may be also related to spine instability; in case of RT, it has been shown that degree of instability resulted to be predictive for pain response after radiotherapy, as a lower instability corresponds to a better pain control after RT [33]. This may support the hypothesis that pain resulting from mechanical spinal instability responds less well to radiotherapy compared with pain from local tumor activity [5].

However, RT treatment can be associated with various side effects depending on the affected column segment, its extent, delivered radiation dose and radiation delivery technique. The spectrum of symptoms ranges from pharyngitis/sore throat for metastases located in the cervical spine, esophagitis/dysphagia and limited lung fibrosis very rarely progressing to symptomatic pneumonitis in cases of the dorsal vertebrae involvement, gastrointestinal disorders like emesis, diarrhea and abdominal cramps because of the irradiation to the lumbar column, up to exceptional and unforeseeable bystander effects [34,35]. Low blood cell count (anemia, leukopenia or even pancytopenia) may be consequent to RT treatments of large spine targets, such as in half-body irradiation [36]. Moreover, complete resolution of pain are rare, ranging from 10% to 20%, and up to 20% of patients will need reirradiation within 3–6 months because of loss of efficacy of the initial RT or development of metastatic progression [37,38].

More advanced precise RT techniques, such as, in order of complexity, *intensity-modulated radiotherapy (IMRT)*, *volumetric modulated arc therapy (VMAT)* and *stereotactic body radiotherapy (SBRT)*, have been developed over the years to improve the therapeutic index of RT in other challenging scenarios [39,40,41]; from these ones, all the above techniques have been borrowed to increase the radiation dose to the metastatic vertebra without exceeding the tolerance dose of the spinal cord while drastically reducing the radiation exposure of neighboring organs-at-risk (OARs) [42].

In patients with complex target volumes or challenging geometry, *intensity-modulated radiotherapy (IMRT)* has been used to provide highly conformal target coverage while sparing organs at risk with results of bone density and pain control comparable to 3-dimensional conformal radiotherapy (3DCRT) [43,44]. Although overcoming the technical challenges of conventional treatment planning by delivering more conformal doses from several geometric angles, IMRT can significantly increase treatment delivery time because of the multiple beam angles required [45]. This is especially true when using static IMRT (in the step-and-shoot and sliding-window forms) instead of fully rotational techniques (VMAT).

VMAT, which is an evolved form of IMRT, allows an improved dose escalation and a reduction in radiation-induced toxicities and in overall planning time, when compared with the current conventional technique [37].

The *stereotactic body radiation therapy (SBRT)* has gradually emerged as a promising and well-tolerated treatment strategy for delivering high doses of radiation in a dose-escalated manner to the target vertebral region while minimizing radiation to nearby critical structures, such as the spinal cord, and to surrounding normal tissues [46]. It implements the use of radiation delivery by VMAT or non-isocentric techniques (Cyberknife™) with stereotactic equipment for highly precise treatments [47,48,49]. SBRT appears to be useful in pain relieving and, more rarely, neurological improvement, along with improving the local control and minimizing treatment-related toxicities [37,38,43,50,51,52]. This ablative high-dose approach seems to be really effective and should therefore be reserved for a highly-selected group of patients, such those with more favorable prognosis [53] or with radioresistant tumors [54], with oligometastatic disease of the spine [55] or with a previous history of vertebral irradiation [38]. The indications for the SBRT use and its integration with other treatments (i.e., surgery) in spine metastases have been summarized in decision-making algorithms [56] as well as the specific dose-volume constraints to limit the risk of spinal cord and cauda injuries [57]. Moreover, SBRT could offer safe re-irradiation since it can spare adjacent organs at risk, including the spinal cord [50,58]. However, SBRT is more likely to cause adverse-related events such as vertebral compression fracture; this complication is more frequent in case of lytic disease, pre-existing compression and spinal instability [55].

Lastly, new radiation delivery techniques like the Spatially Fractionated ones might be helpful when treating inoperable bulky tumors invading the spine by exploiting their immunogenic effect while not exceeding the spinal cord tolerance [59,60].

#### 4.2.1. Radiotherapy Protocols and Tolerance

In the palliative treatment of spinal metastases overall treatment time of radiotherapy should be as short as possible [42,61]. Moreover, the treatment should be tailored on the patient’s characteristics and the optimum radiation dose, fractionation regimen and treatment time remains debatable [62,63].

Many studies have shown that RT in lower doses is equivalent to higher radiation doses and that short course treatment should be preferred [64,65,66,67,68,69,70,71].

#### 4.2.2. Combination of Radiotherapy and Other Treatments

The therapeutic approach for patients with spinal metastases should be decided on a case-to-case basis; for this reason, many patients receive radiotherapy alone, while others can benefit from the associations with other surgical and non-surgical treatments.

In particular, in has been shown that the combination of RT and bisphosphonates in case of metastatic renal cell carcinoma may reduce the odds of pathological fractures and spinal cord compression and the combination of RT and PD-1 inhibitor may positively influence pain control and neurological improvement [72,73].

In those cases addressed to surgery, it has been proven that *RT following surgery* for spinal cord decompression and vertebral fixation or reconstruction has been proven as effective combined treatment for MESCC in case of patient with good prognosis and overall performance status and *radiofrequency ablation (RFA)* followed by RT may ensure a better local and pain control than RFA or RT alone [74,75].

As regard physical therapies, the isometric paravertebral muscle training (IPMT) following RT has been proposed as a feasible treatment which may improve mobility, pain control and QoL in patients with unstable spinal metastases [76].

As regard pain therapy, RT combined with *tapentadol* may improve pain and patient’s QoL thanks to the improvement in emotion functioning and fatigue due to tapentadol double action as opioid agonist and noradrenalin reuptake inhibitor [77].

### 4.3. Ablative Surgery

#### 4.3.1. Radiofrequency Thermal Ablation (RFA)

*Radiofrequency ablation (RFA)* is a minimally-invasive ablative technique which uses an alternating current through in order to ablate the tumor through the heating of an electrode; once the critical temperature is reached, local tissues and cells undergo thermal coagulation, causing necrosis of the tumor [78]. RFA was firstly used for the treatment of osteoid osteoma in 1992, and since then, successful ablation of neoplastic lesions and for painful osteolytic metastases has been reported more and more frequently [79,80].

The clinical goal of RF ablation in vertebral metastases is primarily pain reduction, by the destruction of local sensory nerve fibers [81]. Since the introduction of bipolar RFA system, the flow of the current is more predictable and precise than older monopolar systems [3]. This approach allows for a regular intraprocedural clinical examination and may help avoid possible surrounding neural damage, such as spinal cord injury. Immediate pain relief is observed with subsequent improved QoL [3]. Thus, the possibility of treating large volumes adjacent to vulnerable structures individually defined by the chosen electrode array can be performed [82].

Moreover, RFA is thought to decrease the volume of the metastatic lesion and inhibit osteoclast activity [75].

RFA is a mini-invasive treatment which presents several advantages, since it can be repeated several times with no risk of biological exposure than RT [3] and it provides rapid and long-lasting significant pain relief in patients with painful metastatic bone tumors [79,80,81,83].

The Metastatic Spine Disease Multidisciplinary Working Group stated that RFA may be ineffective in treating bone metastases in some situations: asymptomatic spinal metastases in patients with poor general condition, life expectancy of less than 6 months, pathological vertebral compression fracture and epidural spinal cord compression [1,56].

#### 4.3.2. Combination of Radiofrequency Thermal Ablation and Other Treatments

Several studies have evaluated the combination of RFA with *percutaneous vertebroplasty (PVP)* or with *balloon kyphoplasty* (BKP) as safe and effective treatment for spinal metastases. RFA causes coagulation necrosis of tumor cells and the tumor cavitation prior to vertebral augmentation, thus reducing the complications of cement leakage and improving quality of life [78,81]. On the other hand, the addition of cement augmentation provides mechanical stability and pain relief in cases of osteolytic metastatic lesions or those associated with pathological fractures; since the cement is highly resistant to compression forces, it is suitable for fractures involving weight-bearing bones like vertebral body [82]. As suggested by more recent multidisciplinary guidelines, RF ablation with vertebral augmentation has become one of the ideal treatment modalities that may be performed alone or in combination with radiation therapy in the treatment of metastatic bone disease [81].

#### 4.3.3. Other Ablative Techniques

Another technique which employs thermal ablation for inducing coagulative necrosis of the tumor is *microwave ablation (MWA)*. Compared with RFA, MWA is relatively insensitive to the high impedance of bone and desiccation, allowing deeper thermal penetration and more efficient heating; furthermore, MWA facilitates the use of higher temperature, a wider range, and a shorter time of ablation; however, in the treatment of spinal metastases, rapid heating and large ablation range may be disadvantageous, since overheating the tissue surrounding the tumor could result in serious neurologic complications [84].

The combination of MWA and *vertebral augmentation* has been proven as safe and feasible treatment for painful bone metastases, relieving pain and increasing bone stability.

*Cryoablation (CA)* may be a minimal invasive treatment for patients with spinal tumors not responding to RT [10]. CA can be used alone or combined with *systemic* or local therapies such as *RT* and *vertebral augmentation*. CA results in fast and long-lasting pain relief as well as acceptable rates of local tumor control. Main limitations of this technique are the limited experience and the risk of fracture [85].

However, ablative techniques such as RFA or CA, are contraindicated for tumor location within 1 cm of important structures such as the spinal cord, major nerves, and blood vessels [1,56].

### 4.4. Augmentation Surgery

Surgery remains a fundamental option in the therapeutic armamentarium of treatments for spinal metastases.

However, currently there is no consensus on the most effective treatment for the treatment of spinal metastases aimed to improve patient’s QoL nor on the indication for the type of surgical intervention; in fact, surgery if often urgently indicated for sudden neurological impairments and a pre-operative multidisciplinary evaluation based on objective score is not always possible [86,87].

In case of pathological fracture secondary to spinal metastases, surgery may rely on two different treatments: *open spinal surgery* and *percutaneous spinal surgery.* As regard open surgery, *metastasectomy* may play an important role in effective local control of tumor in selected patients; however, it may delay further RT. In order to improve patients’ outcome, adjuvant therapy with both *targeted therapy* and *cytokine therapy* may positively affect patient’s survival [88].

Percutaneous spinal surgery (*spinal fixation* or *vertebral augmentation*) is associated with less morbidity than conventional open surgery for restoration of spinal stability. Preservation of the paraspinal musculature limits postoperative pain and hospital stay, and percutaneous spinal surgery does not involve delay in radiotherapy; moreover, percutaneous surgery may allow for rapid improvement in quality of life and walking ability for patients with spinal instability [8].

At present, vertebral augmentation (*vertebroplasty and kyphoplasty*) is considered to be the preferred palliative surgical treatment for patients with spinal metastases [89]. Besides the stabilizing effect of bone cement, the heating of cement polymerization leads to tumor necrosis [90].

#### 4.4.1. Percutaneous Vertebroplasty (PVP)

*Percutaneous vertebroplasty (PVP)* was first introduced as a treatment option for aggressive hemangiomas of the lumbar spine [91]. Over the years, PVP has been used for the treatment of painful vertebral metastases, with great efficacy in relieving pain, restoring spine stability and improving patient‘s QoL. In cases of preservation of posterior wall of the vertebral body, PVP represents a less invasive alternative to vertebral replacement [90]. However, PVP may entail local complications; in particular, *local bone destruction progression (LBDP)* may occur early in case of reduced filling rate and cement volume; whereas filling rate > 0.646 was predictive of late LBDP [92]; whereas, complete vertebral filling may increase the odds of cement leakage and adjacent vertebral fractures [89].

#### 4.4.2. Balloon Kyphoplasty (BKP)

Recent reports have shown promising results of *balloon kyphoplasty (BKP)* for spinal metastasis. BKP is used to restore vertebral height through an inflatable balloon to reduce the pressure of cement injection and the risk of leakage. This technique seems to be associated with low postoperative complications as well as significant decrease in pain, use of opioids and length of hospital stay [91]. BKP main indications are painful or lytic vertebral metastases and absence of posterior wall defect. 

BKP is performed through injection of polymethylmetacrylate (PMMA), even if some authors investigated the use of VK100, a mixture of Dimethyl Methylvinyl siloxane and Barium Sulphate, in order to reduce some disadvantages, such as exothermic reaction, rapid solidification and absence of osteoconduction [93].

#### 4.4.3. Open Kyphoplasty 

Kyphoplasty may be performed also with open surgery. The *open kyphoplasty (OKP)* is performed through decompressive hemilaminectomy associated with a contralateral percutaneous kyphoplasty, and in some cases to a posterior fixation, for symptomatic metastatic lesions in selected patients [94].

### 4.5. Spinal Cord Stimulation (SCS)

Considering the more recent field of interest in alternative interventional free-of-side-effects treatment for cancer-related pain, the clinical effectiveness of *spinal cord stimulation (SCS)* has been recently investigated in other chronic painful conditions than failed back surgery syndrome (FBSS) [95,96]. The literature regarding SCS for the treatment of cancer-related pain is largely represented by case reports and small case series [97,98,99]. Some authors advocated SCS as effective treatment for refractory chemotherapy-induced peripheral neuropathy. Moreover, many patients with primary spinal tumors or metastasis may require spinal surgery and vertebral fixation with the subsequent risk of developing post-surgical FBSS-like back pain. Therefore, it is suggested that SCS should be considered early in the treatment algorithm for these patients with both post-surgical lumbar and cervical radicular pain [98,100]. The latest evidence about SCS in cancer-related pain suggested that SCS may be a valuable and effective technique for the treatment of refractory cancer pain and chemotherapy-related pain [98].

## 5. Conclusions

The spine is the most common site of metastatic bone tumors, which often require a prompt treatment in order to improve patient QoL. Since the short life expectancy in case of spinal metastases, patients are often addressed to palliation. In the wide scenario of palliative treatment, nowadays recent medical or surgical techniques represent valuable options for ameliorating pain and improving patients QoL in such condition.
